# Supplementary material for: Optimization of ship hull forms by changing CM and CB coefficients to obtain optimal seakeeping performance
Source: PLoS One. 2024 May 6;19(5):e0302054. doi: 10.1371/journal.pone.0302054 (PMC11073696; doi:10.1371/journal.pone.0302054)
Supplement: Attachment — Submitted filename: reply to reviewers-R1.docx [file pone.0302054.s001.docx]

**Dear Respected Editor,**

We are very thankful to editorials and referee #1 for their useful comments and suggestions on our manuscript. We have revised the manuscript accordingly and some of the detailed corrections and revisions are listed as follows:

With my best regards,

Hassan Ghassemi

**=================**

**Reviewer #1**

Q1:The paper appears to suggest an application of a standard optimization method. It is not clear to me what is the engineering science-based contribution in terms of optimization methods and seakeeping. Is this simply an application? What is the originality and the ultimate goal?

R1: Thanks for your comment. The key novelties of this work are: (1) using block and midship coefficients as optimization parameters to improve seakeeping and (2) developing a fuzzy model to generate hull form variations while keeping other parameters constant. Unlike previous hull form optimization studies that use complex geometrical parameters, we use only CB and CM as variables in a novel fuzzy hull generation model to provide useful insights.

2. Literature survey in section 1 should be put in a new section and a table highlighting the state of the art should be displayed. The introductory section should expand to make clear my questions under (1.) above. Similarly the conclusions and highlights of the paper should be updated.

3. You should add a flowchart highlighting the seakeeping optimisation procedure proposed in section 2. the flowchart should display the elements of originality of your method.

R3: This flowchart is added to the manuscript.

4. I am not of the opinion that the results and discussion sections display anything more or less than the obvious. The in depth processing of results, the impact in terms of engineering science and design are not evident.

R4: more discussions are added to the manuscript.

===========================

**Response to the comments of Reviewer #2:**

Q1-1: Limited Methodology Explanation:

While the summary mentions the use of a fuzzy model and multi-objective optimization, a brief explanation of how these methodologies were applied in the study could enhance clarity for readers unfamiliar with these techniques.

R1-1: The seakeeping analysis was performed using strip theory-based MAXSURF software and the optimization procedure was performed using genetic algorithm.

Q1-2: Detail on Other Motion Indices:

The summary briefly mentions that heave and roll motions, as well as added resistance, were not significantly influenced. Adding a bit more detail on these aspects could provide a more comprehensive understanding of the study's findings.

R1-2: Thanks for your comment. The following sentence is added to the abstract:

Heave and roll motions, as well as the added resistance, were not significantly influenced by the coefficients of $C_{M}$ and $C_{B}$. However, increasing the hull form parameters increases the maximum RAOs of heave and roll motions.

Q1-3: Quantitative Results:

The summary could benefit from the inclusion of specific quantitative results, such as numerical values or percentages, to provide a more concrete understanding of the study's outcomes.

R1-3:

Q1-4: Conclusion Impact:

The summary mentions the selection of and based on seakeeping performance but does not elaborate on the broader implications or potential applications of these findings. Including a brief conclusion, impact section could enhance the summary.

R1-4: These findings can provide useful insights for shipbuilding technology for producing more compatible vessels for specific seaways. The following sentence is added to the abstract.

These findings might be used by shipbuilders to construct the vessel with more efficient seakeeping performance.

Q2-2: Limited Explanation of Seakeeping Indices:

The summary introduces seakeeping indices such as heave, pitch, roll motions, added resistance, and motion sickness but does not provide a detailed explanation of these terms. Adding a brief definition or context could improve reader understanding.

R2-3: There are six degrees of freedom on each vessel, including three linear motions (surge, sway, heave) and three angular motions (roll, pitch and yaw). Also, a detailed description of motion sickness incidence is provided.

The sea-going ship that operates in open waters rarely sails in calm weather. On the contrary, ship's behaviour at sea is often affected by waves and wind. When a ship navigates in a seaway, the ship′s forward speed decreases, compared to that in calm sea, because of added resistance due to winds, waves, rudder angle, and so forth. The magnitude of added resistance is about 15–30% of calm-water resistance (Seo et al., 2013). The immediate effect of waves is ship’s motions and accompanying phenomena, such as accelerations. Ship accelerations, in turn, particularly vertical ones, impact on the human body and may cause motion sickness. The term “motion sickness”, on ships known as sea sickness, is understood as a sickness due to ship motions that results in physical discomfort, with such symptoms as irregular breathing, nausea, vertigo, paleness and vomiting. In extreme cases a passenger or crew member has to be transferred to hospital. The actual reason for sea sickness is lack of conformity between different stimuli, eye signals and the labyrinth (inner ear), received by the human brain. People mainly suffer from sea sickness under deck, where the eye does not register any stimuli that the labyrinth would interpret as motion (Cepowski, 2012).

Despite scientific observations and research, no exact relations have been determined between ship motions and motion sickness. McCauley and O’Hanlon estimated quantitatively the impact of ship motions on the percentage of people that would suffer from sea sickness. It turned out that vertical accelerations in particular were responsible for motion sickness, while rolling and pitching had slight influence. Additionally, it was found that at a frequency of 0.167 Hz the occurrence of motion sickness increased. The MSI index (Motion Sickness Incidence) is commonly used for assessing possible occurrence of the illness:

$$MSI=100\left[ 0.5\pm erf\left( \frac{\pm{log}_{10}\frac{a_{v}}{g}\pm\mu_{MSI}}{0.4} \right) \right]$$

where MSI is the motion sickness incidence index, erf is the error function, $a_{v}$ is the mean value of vertical accelerations at a selected point and $\mu_{MSI}=-0.819+2.32\left( {log}_{10}\omega_{E} \right)^{2}$.

Q2-3:

Need for Context on Fuzzy Model:

While the use of a fuzzy model is mentioned, there is no information on why this specific modeling approach was chosen or how it contributes to the study's objectives. Providing a brief context or justification for the use of the fuzzy model would strengthen the summary.

R2-3: Thanks for your comment. The following paragraph is added to the revised manuscript:

The **Preference Ranking Organization METHod for Enrichment of Evaluations** that is used in this study has particular application in decision making, and is used around the world in a wide variety of decision scenarios, in fields such as business, governmental institutions, transportation, healthcare and education. Rather than pointing out a "right" decision, the Promethee method helps decision makers find the alternative that best suits their goal and their understanding of the problem. It provides a comprehensive and rational framework for structuring a decision problem, identifying and quantifying its conflicts and synergies, clusters of actions, and highlight the main alternatives and the structured reasoning behind. we can cite applications to portfolio and stock selection problems (Albadvi et al., 2006; Marasović & Babić, 2011; Vetschera & De Almeida, 2012), to environmental issues (Hermans et al., 2007; Ilić et al., 2011; Nikolić et al., 2009) to energy management (Ghafghazi et al., 2010; Madlener et al., 2007) and shipping industry (Ahmadi & Herdiawan, 2021; Animah & Shafiee, 2021; Emovon, 2016; Glavinović & Vukić, 2023; Zheng et al., 2023).

Weaknesses:

Q3-1: Limited Wave Amplitude Range: The presented validation focuses on a specific wave amplitude (1.5 cm). While this provides valuable insights into the model's accuracy under these conditions, it would be beneficial to assess its performance across a broader range of wave amplitudes to ensure its robustness.

R3-1: Thanks for your comment. The vessel considered in this study is planned to sail in the Gulf of Oman. This wave amplitude was selected based on the significant wave height typically encountered by the ship in the Gulf of Oman.

Q3-2: Assumption of Similar Wave Lengths: The emphasis on extreme motions when the wave length is almost the same as the vessel length is noted. However, the model's performance under conditions where this similarity doesn't hold may require further investigation. The model's limitations under diverse wave conditions should be acknowledged.

R3-2: Thanks for your comment. The considered scenario was chosen based on the extreme condition that might be encountered by the vessel, i.e., the resonance condition. Performing extra simulations and presenting the results will lengthen the paper.

Q3-3: Need for Additional Validation Scenarios: While the presented validation results are promising, conducting validations under various conditions, including different vessel speeds and wave frequencies, would further strengthen the model's credibility.

R3-3: Thanks for your comment. In its current form, the paper is lengthy. We think that performing extra validations and presenting the results leads to at least 1-2 extra pages.

Q5-2: Specific Speed: The analysis is conducted at a specific speed (35 knots), limiting the generalization of findings to other speeds.

R5-2: Thanks for your comment. Actually, as pointed out in the previous comments, this study was conducted on a patrol vessel optimized for sailing in the Oman Sea at a certain speed.

Q5-3: Complexity of MSI: While the study identifies the impact on MSI, the complexity of motion sickness requires further investigation for a comprehensive understanding.

R5-3: Thanks for your fruitful comment. We will study this issue in our future studies.
