# Supplementary material for: Optimization of ship hull forms by changing CM and CB coefficients to obtain optimal seakeeping performance
Source: PLoS One. 2024 May 6;19(5):e0302054. doi: 10.1371/journal.pone.0302054 (PMC11073696; doi:10.1371/journal.pone.0302054)
Supplement: Attachment — Submitted filename: reply-R2.docx [file pone.0302054.s002.docx]

Q1: Clarify the acronym RAO when it is introduced in the abstract.

R1: Thanks for your comment. RAO is defined in the revised version of abstract.

Q2: Ensure that all technical terms, such as hull coefficients (, ), are consistently defined or referred to in the article.

R2: … including water plane coefficient ($C_{wp}$) and prismatic coefficient ($C_{p}$) …

… The body lines generated by $C_{M}$ and $C_{B}$ in this model vary indecently, but do not affect the other geometric parameters ($C_{P}$, $L$ (Length) and $B$ (Breadth))…

Q3: The introduction is informative, but consider briefly summarizing the main findings and contributions of your study at the end of this section.

R3:

The following paragraph is added to the manuscript:

It will be shown that by optimizing these coefficients, pitch and MSI will improve. On the other hand, the effect of these coefficients on the roll and heave motion as well as the added resistance is negligible.

Q4: Mention if there are any limitations or gaps in previous research that your study aims to address.

R4: This comment is already mentioned in the manuscript:

Unlike previous hull form optimization studies that use complex geometrical parameters, we use only $C_{B}$ and $C_{M}$ as variables in a novel fuzzy hull generation model to provide useful insights.

Q5: Provide more details on the fuzzy model used to generate the hull form. How does it work, and what are its advantages in this context?

R5: Thanks for your comment. This issue is adderessed in the manuscript.

Modeling Fuzzy changes in desired parameters

Fuzzy logic has advantages in ship hull optimization, including:

- Handling imprecise and uncertain data: Fuzzy logic effectively deals with imprecise and uncertain information, common in ship design and optimization.
- Flexibility: Fuzzy logic incorporates subjective human knowledge and expertise into the optimization process, making it adaptable to different design requirements.
- Non-linear relationships: Fuzzy logic models complex, non-linear relationships between design parameters and performance criteria, which traditional optimization methods may struggle with.
- Robustness: Fuzzy logic-based optimization methods are often more stable, handling variations and uncertainties in design parameters without significantly impacting results.

This paper presents a fuzzy structure model for midship coefficient changes. The variation of these coefficients does not affect the other geometric parameters. Changes should be applied in such a way as not to affect the volume of the vessel. It is, therefore, necessary to develop a mathematical model of the changes that will cause the exact change in the desired parameter while maintaining other geometric parameters constant while holding constant the volume.

A fuzzy membership function, which is an extended Bell membership function, is defined as a function that makes changes to the body's lines. As a result, if any other changes are made, it increases and decreases the same volume, thus maintaining a constant volume change. Bell function is expressed as follows and its shape is a modified Gaussian distribution. Figure 3 illustrates the distribution shape of the Bell function.

$$Bell\left( x;a,b,c \right)=\frac{1}{1+\left| \frac{x-c}{a} \right|^{2b}}$$

(6)

Figure 3. Bell’s membership functions.

Figure 3 illustrates how the fuzzy function at the upper level makes good changes to the body line in the middle and returns the variant body lines to the original lines at both ends with instant cuts in both directions, with the bell acting as a coefficient of variation. It is now necessary to restore the taken area of one side to the opposite direction using the same function used in the interval (0, -1). Therefore, this function is multiplied by a sine function. The fuzzy function coefficients in this model are defined as follows:

| $G\left( x \right)=gbellmf\left( x1,\left[ 3 4 0 \right] \right)$  $a=3$, $b=4$, $c=0$. | (7) |
| --- | --- |

The following is a description of the effects of parameters on the function:

Coefficient $b$: Coefficient $b$ is a positive number and is usually considered to be 4. The number corresponds to the upper part of the curve and contributes to the flat portion of it. This coefficient has a significant impact on the performance of the function. Figure 1 illustrates the changes in the crude bell membership function.

Coefficient $a$: Based on mathematical calculations and modeling, a coefficient equals 1.44 times the length of each line within a body. There will obviously be a difference in the coefficient of the body lines of the vessel.

Coefficient $c$: It determines the center of the bell function that is considered equal to the half-length of each line.

The sine function of the fuzzy function multiplied by Bell is given below:

$$F\left( x \right)=\sin\left( \frac{x-\min(x)}{\max\left( x \right)-\min(x)} \right)$$

(8)

Data set x represents the length of the body lines to a point at which the fuzzy shift applies the same area to the other side. The length of the new points is determined by the function $H (x)$. Using coefficient $L$ at the end of the function, the different sizes of the changes are applied manually. This is the fuzzy function - neurological function obtained from Bell's model to apply to the vessel model:

$$H\left( x \right)=\left[ G(x)\times F(X)\times L \right]\left[ \frac{x}{\max\max\left( x \right)-\min(x)}+\min(x) \right]$$

$$H\left( x \right)=\left[ \left[ \frac{1}{1+\left| \frac{x}{3} \right|^{8}} \right]\times\left[ \sin\left( \frac{x-\min(x)}{\max\max\left( x \right)-\min(x)} \right) \right]\times L \right]\left[ \frac{x}{\max\max\left( x \right)-\min(x)}+\min(x) \right]$$

(9)

Using the obtained membership function, a fuzzy inference system is formed using a membership function $H (x)$ and a simple inference function. Fuzzy relations are applied to any of the vessel body lines based on the customization done. As a result of using the above model, the vessel volume is not altered and the coefficient $C_{B}$ and $C_{M}$ are affected positively by adjusting geometrical values.

Q6: Clarify the meaning and significance of the Seakeeping Performance Index (SPI) as the objective function.

R6: The Seakeeping Performance Index (SPI) is a common measure of how well a ship handles rough seas. It calculates the percentage of time that the ship stays within specific motion limits. The SPI depends on assumptions about the frequency of different sea states and the likelihood of different ship speeds and headings. To evaluate the SPI, we predict how the ship will move for each combination of heading, speed, and sea condition. We then compare these predictions to a set of criteria that determine the optimal performance limits for the ship's mission in that particular sea condition.

Q7: The literature review is comprehensive, but consider grouping the studies by themes or methodologies to enhance readability.

R7: thanks for your comment. This is already performed in the manuscript:

Table 1 Relevant studies on ship hull form optimization.

| ***Method*** | ***Conducted by authors*** |
| --- | --- |
| Thin-ship strip theory | A. Day & Doctors, 1996; A. H. Day & Doctors, 2001; Hsiung, 1981). |
| Slender-ship approximation | (Yang et al., 2001 |
| Fourier-Kochin flow method | Noblesse, 2001; Noblesse & D, 2000 |
| Strip theory | B. S. Kim et al., 2020, 2021; Niklas & Karczewski, 2020; Rosa et al., 2021 |
| Potential-flow panel methods based on Rankine sources | Campana et al., 1999; Choi et al., 2015; Dejhalla et al., 2001; C.-H. Huang et al., 1998; Lu et al., 2016; Lv et al., 2013; Mittendorf & Papanikolaou, 2021; Peri et al., 2001; Saha et al., 2004; Vu & Nguyen, 2020. |
| Boundary element method | Esmailian et al., 2017; Ghassemi & Zakerdoost, 2017; Zakerdoost et al., 2013; Zakerdoost & Ghassemi, 2018, 2019, 2021, 2023 |
| CFD | Campana et al., 2006; Coppedè et al., 2019; Han et al., 2012; He et al., 2011; Hino, 1998, 1999; F. Huang et al., 2015; F. Huang & Yang, 2016; Jeong & Jeong, 2020; H. Kim et al., 2008, 2010, 2012; H. Kim & Yang, 2010; Nazemian & Ghadimi, 2021, 2022b, 2022a; Serani et al., 2022; Tahara, 1998, 2004; Tripathi & Rajagopalan, 2023; Wang & Kim, 2023; Zhang et al., 2018 |

Q8: Consider presenting the results in a more structured manner, perhaps using subheadings for different aspects of the study.

R8:

Q9: Discuss the practical implications of the optimized and coefficients for ship design and seakeeping performance.

R9: Naval architects and marine engineers use advanced computational fluid dynamics (CFD) simulations and optimization techniques to calculate and optimize these coefficients for a specific ship design. They can minimize resistance, improve maneuverability, reduce slamming and motions in rough seas, and enhance overall seakeeping performance.

Q10: Summarize the key findings concisely in the conclusion section and emphasize their significance for the field of ship design and optimization.

- R10: The pitch motion can be uncomfortable for passengers and crew and can also affect the stability and performance of the vessel. Hence, decreasing this motion is of great importance in ship design.

Decreasing MSI will improve the conformability of the crew onboard the vessel.
